# Supplementary material for: Real-time terahertz imaging with a single-pixel detector
Source: Nat Commun. 2020 May 21;11:2535. doi: 10.1038/s41467-020-16370-x (PMC7242476; doi:10.1038/s41467-020-16370-x)
Supplement: Supplementary file 1 — Supplementary Information [file 41467_2020_16370_MOESM1_ESM.pdf]

Supplementary Information: Real-time terahertz imaging with a single-pixel detector

Rayko Ivanov Stantchev[1,*], Xiao Yu[2,3], Thierry Blu[1], and Emma Pickwell-MacPherson[1,3]

[1]Chinese University of Hong Kong, Electronic Engineering, Hong Kong SAR
[2]State Key Laboratory of Power Transmission Equipment & System Security and New Technology, School of Electrical Engineering, Chongqing University, Chongqing 400044, China
[3]University of Warwick, Physics, Warwick, CV4 7AL, UK
[*]rayko@cuhk.edu.hk

Supplementary Note 1: Raw THz pulses

In supplementary figure 1 we show the raw THz pulses as measured by our system (Menlo K15 THz spectrometer), with the colour of the line indicating the optical pump fluence and the black lines being our reference THz pulses without any optical excitation. This is the raw data used to create the experimental results in figure 1. The top, middle and bottom set of pulses being respectively measured in the transmission, TIR and reflection geometries shown in figure 1 of the main work. Note that the transmission and reflection data sets have been vertically offset for visual clarity. As briefly discussed in the main work, the reflection geometry creates a big preliminary pulse that is not modulated by the photoexcitation of the silicon. Only the pulses that reach the top wafer interface are modulated. The TIR geometry also has a small pre-pulse, which is caused by the fact that we placed a silicon wafer on top of the silicon prism. This creates a small air-gap in between the silicon-silicon interface which generates this small pre-pulse. For a fair a comparison, we placed the same wafer in the transmission and reflection measurements since our silicon prism had a different carrier life-time giving a very different modulation depth.

Supplementary Note 2: Drude modelling

The dielectric response of photoexcited silicon can be adequately described by the Drude model[1–3]:

$$\varepsilon(\omega) = \varepsilon_b - \frac{\omega_p^2}{(\omega^2 - i\omega/\tau_c)}, \qquad \omega_p^2 = \frac{Ne^2}{m} \tag{1}$$

where $\varepsilon_b = 11.9$ is the background dielectric constant of silicon due to the bound electrons, ω_p is the plasma frequency, N is the density of free charge-carriers, m is their effective mass ($m = 0.26m_e$[4]) and e is the electric charge and τ_c is the average carrier collision time ($160fs$ for undoped silicon[4]). We modulate our THz via optical excitation and only considering the free carrier generation and linear recombination in the wafer, the number of charge carriers in the equilibrium state at depth z is be given by

$$N_{si}(z) = \frac{I_0(1-R)\tau_l e^{-z/d}}{2\hbar\omega_l V}, \tag{2}$$

where I_0 is the incident intensity, R is reflectivity of silicon at the pump wavelength, tau_l is the carrier lifetime (estimated to be $60\mu s$ from figure 2a), V is the volume where the carriers are confined within, $\hbar\omega_l$ is the pump-photon energy and d is the diffusion length of the charge-carriers. We photoexcite an area much larger than the THz-beam thus $V = Ad$, where A is the area of our THz-beam. The charge carriers diffuse inside the wafer as described by the 3D diffusion equation, hence their mean square displacement is

$$< x^2 >= 6D\tau_c, \tag{3}$$

where D is the diffusion coefficient given by the Einstein-Smoluchowski relation $D = \mu_q k_B T/q = \tau_c k_B T/m*$ for carrier mobility $\mu_q = q\tau_c/m*$. supplementary equation 3 gives values around $0.5mm$ which are lengths comparable in size to the THz-wavelengths, especially inside the silicon, therefore we need a stratified multi-layered model to account for the depth distribution of the charge carriers. To this end, we use the transfer matrix method[2,5]. This theory states that the transmission and reflection functions of our system is[2,5]

$$t = \frac{2q_i}{q_iM_{11} + q_fM_{22} + q_iq_fM_{12} + M_{21}} \tag{4a}$$

$$r = \frac{q_iM_{11} - q_fM_{22} + q_iq_fM_{12} - M_{21}}{q_iM_{11} + q_fM_{22} + q_iq_fM_{12} + M_{21}} \tag{4b}$$

where $q_{i,f} = n_{i,f}\cos\theta_{i,f}$ for $n_{i,f}$ being the refractive indices of the initial and final media, respectively with incident and final angles $\theta_{i,f}$, enclosing the multilayer system and M is a 2×2 matrix associated with the propagation through the entire multilayer system. This matrix is given by the product of the individual layer matrices, $M \equiv M_1M_2M_3...M_N$, describing the propagation through each layer. The characteristic matrix of the jth layer, M_j, with thickness l_j and dielectric function ε_j is given by

$$M_j = \begin{bmatrix} \cos\beta_j & \frac{i}{q_j}\sin\beta_j \\ iq_j\sin\beta_j & \cos\beta_j \end{bmatrix}, \tag{5}$$

where $\beta_j = \omega l_j n_j \cos\theta_j/c$ is the phase delay associated with light propagation inside the jth layer.

When using this multilayer approach, this means that in supplementary equation 2 $V = Al_j$ when calculating the carrier densities in each layer. After knowing the carrier densities one can calculate the permittivities of each layer via supplementary equation 1 and then calculate the final transmission

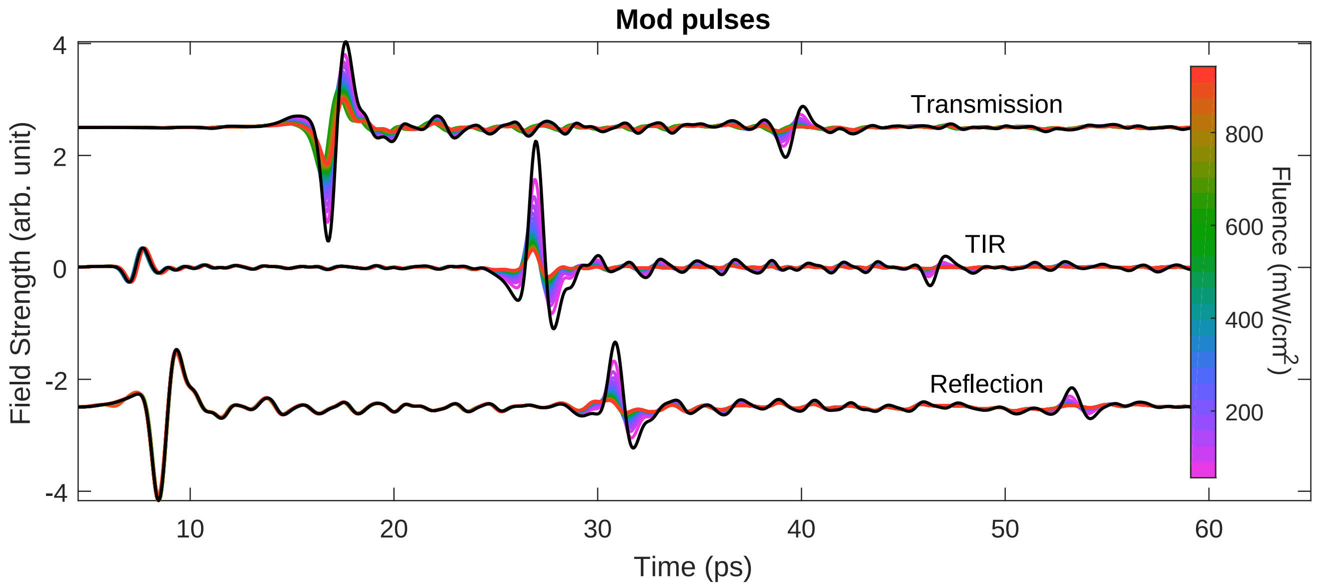

Supplementary Figure 1. Raw THz pulses These are our THz pulses. The Transmission and Reflection data have been vertically offset for visual clarity.

and reflection coefficients of the system. Note: attention is needed to account for the losses at the first interface in the reflection geometry (we assume that only the power transmitted into the wafer is modulated).

Supplementary Note 3: Fast estimation of mask values

As mentioned in the main work, we continuously measure a THz signal whilst changing the projected mask at regular period T_p and the temporal response of each individual mask is given by eq. 3. This means that our total signal is given by

$$y(t) = \sum_{j=0}^{N-1} B_{j+1}(1 - e^{-(t-jT_p)/\tau}) + B_j e^{-(t-jT_p)/\tau}, \quad (6)$$

where N is the number of masks we have projected. Our job is to find all the B values that describe the amplitude of each temporal segment describing each displayed mask, where B_0 is the amplitude before projecting the masks. To calculate the first two constants, B_0 and B_1, we need to select the time values from $y(t)$ for which the first mask was projected. This is expressed as a column vector $T_1 = (t_1; t_2; ...; t_{p-1}; t_p)$ where p is the number of samples during each period. Then we have that $y(T_1) = B_1(1 - e^{-T_1/\tau_l}) + B_0 e^{-T_1/\tau_l}$, which can also be expressed in matrix notation as

$$y(T_1) = [f_1(T_1)\ f_0(T_1)] \times \begin{bmatrix} B_1 \\ B_0 \end{bmatrix} \quad (7)$$

where $f_1(t) = 1 - e^{-(t-jT_p)/\tau_l}$ and $f_0(t) = e^{-(t-jT_p)/\tau_l}$ with $j = 0$ for the first period. Then one can just use the least-squares solution to find B_1 and B_0 from this matrix equation. However, one can further find the rest of B values in a single matrix operation. This is based on the fact that $f_1(T_1) = f_1(T_2) = ... = f_1(T_n)$ which can be seen from the $-jT_p$ factor that shifts the exponential in the time domain for each each mask. With this in mind, then we have that

$$[y(T_1)\ ...\ y(T_N)] = [f_1(T_1)\ f_0(T_1)] \times \begin{bmatrix} B_1 & ... & B_N \\ B_0 & ... & B_{N-1} \end{bmatrix}, \quad (8)$$

where $T_{1,2,...,N}$ are column vectors. This is just linear system of equations for which many efficient calculation techniques exists.

In MATLAB this is can be solved using the *mldivide* function, and doing this for the 32×32 images in figure 3 takes less than $30ms$ using MATLAB R2016a on an Intel Core i3-4160 3.6GHz CPU running Ubuntu 19.10 with 12GB of RAM.

Supplementary Note 4: Signal to noise definition

The definition of signal to noise used in section 1.2 of the main work is defined as the mean value of an image area with signal divided by the standard deviation of an area without signal,

$$\text{SNR} = \frac{\text{Mean}(X_{signal})}{\text{Std}(X_{noise})}. \quad (9)$$

In supplementary figure 2 we show the regions that we have defined as ‘Signal’ (‘Noise’) in the red (green) rectangles. These same regions have been used to evaluate the SNRs shown in the figure 3 of the main work.

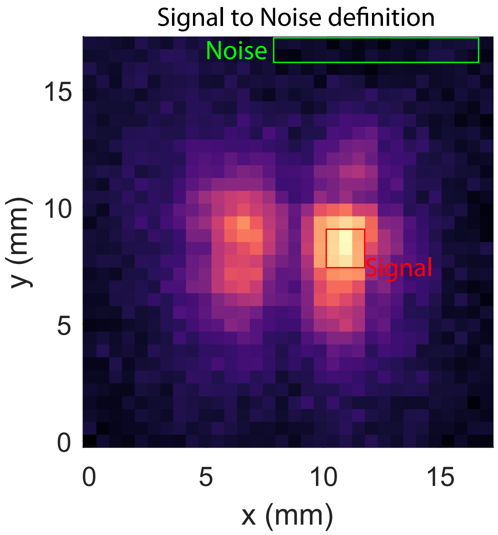

Supplementary Figure 2. The Signal-to-noise in figure 3 of the main work is defined as the mean of the pixels in the red-region (labelled as 'Signal') divided by the standard deviation of the green region (labeled as 'Noise')

Supplementary Note 5: Sine-wave carrier modulation

Noise reduction is of the utmost importance in regards to fast acquisition of good quality images. In THz-TDS systems lock-in amplifiers are routinely used for noise-reduction, however this might result in adding great complexity and cost without improving the image acquisition rate. Most notably, if the slowest component of the THz system is the detector response time, then a lock-in would require a bias-modulation with a frequency slower than the detector-response time thus adding extra measurement time. In the case that the mask modulation is the slowest component of the system, as is the case in this work, then it is conceivable to use a lock-in amplifier. However, the readout rate would have to be ~ 10 times quicker than the slowest component, for accurate fitting estimates, which in turn requires a source modulation rate $5-10$ times quicker than the readout rate, since lock-in amplifiers multiply the input by a reference sine-wave and integrate over a few periods. This, even at $20kHz$ mask modulation-rate already places a stringent requirement of a $>1MHz$ source modulation-rate which is accompanied by extra electrical design considerations.

Accounting for a carrier-wave modulation can be done in post-processing. This would still grant the benefits of carrier-wave whilst adding minimal computational and electrical complexity. Most notably, supplementary equation 6 is multiplied by a sine wave $\sin(\omega t+\phi)$, or a square-wave, of a known frequency ω and phase ϕ. In principle, this carrier frequency can be slower than our mask switch-rate; however, if it is quicker then we can account for low-frequency noise sources since our sine-wave should oscillate around a central zero-value. Any deviation from the central value can be due to source noise and/or temperature fluctuations in electronics, for example, and is easily accounted for by subtracting the offset from the central oscillation value. Further, it needs to be mentioned that the period of the carrier-wave should be a multiple of the mask switch-period since this reduces computational times and is now discussed.

To mathematically describe the application of a carrier-wave in the context described in section Supplementary Note 3:, we have multiplied the right-hand side of supplementary equation 6 by $\sin(\omega t+\phi)$. Therefore, $f_1(t)$ and $f_0(t)$ become $f_1(t) = \sin(\omega t+\phi)(1-e^{-(t-jT_p)/\tau_l})$ and $f_0(t) = \sin(\omega t+\phi)e^{-(t-jT_p)/\tau_l}$. If the period of the carrier wave is a multiple of a mask switching period, then $\sin(\omega t+\phi) = \sin(\omega t+\phi+jT_p)$ for any integer j, thus in this case we still have $f_1(T_1) = f_1(T_2) = ... = f_1(T_n)$ holding true, allowing us to simply solve supplementary equation 8 with our modified definitions of $f_1(t)$ and $f_0(t)$. Adding this sine-wave term to the mathematics only adds about $10ms$ to the computational times in our system. If, however, one cannot apply the correct carrier-wave frequency then it is still possible to obtain all the B values, but one needs to implement a loop and solve supplementary equation 7 for each segment which doubles the computational time on our system. Finally, as mentioned in the previous paragraph, since the carrier-wave oscillates around a zero-central value then one needs to subtract out any low-frequency fluctuations from the raw signals in advance.

In supplementary figure 3**a** we plot the measured signals when applying a sine-wave carrier modulation (red-crosses) and a DC bias (blue-dots) to our THz emitter and the solid lines are the fitted signals. We switch our masks with a period of $1ms$. The images resultant from both methods are shown in fig. 3**b-c**, where the numbers in the top-right corner indicate the SNR. The sine-wave (DC) bias has a SNR of 40.1(18.9), and the reason for this increase is the following: we use a photoconductive THz emitter and detector pair which is a polarization-sensitive system measuring both positive and negative electric field values. Therefore, applying a positive and negative bias to the emitter will cause a reversal of the detected electric-fields. When this effect is exploited, it results in the measurement of positive and negative amplitudes compared to only the positive and zero values achieved by our modulator. As mentioned in the paragraph above, the sine-wave bias also allows for the subtraction of any low-frequency noise offsets caused by laser instability or temperature fluctuations of electronics. The combination of these two effects explains the factor of 2.2 increase in SNR when comparing the images obtained in figs. 3**b** and 3**c**. With increased SNR one can introduce further reductions in acquisition time.

Supplementary Note 6: Hadamard Masks

As stated in the main text, there exist multiple ways of constructing an orthogonal Hadamard matrix. The most common method is called Sylvester's construction and it is as follows.

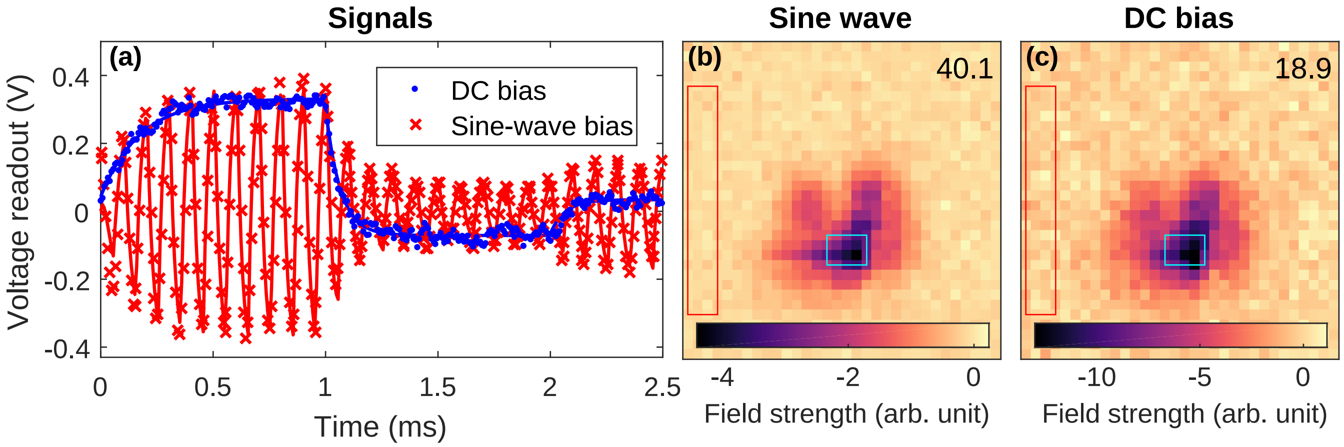

Supplementary Figure 3. **a:** Measured signals when applying a DC (sine-wave) bias to the THz emitter in blue-dots (red-crosses). Lines are the fitted signals with mask switch rate of $1kHz$. **b, c:** The resultant images when using applying a Sine-wave and DC bias, respectively, with the SNR shown in the top-right corner. Note, the signals were acquired by fixing the optical delay line at the maximum of the electric field cycle.

Let H_n be a Hadamard matrix of order n, then

$$H_{2n} = \begin{bmatrix} H_n & H_n \\ H_n & -H_n \end{bmatrix}. \tag{10}$$

Since the lowest Hadamard matrix is $H_1 = [1]$, then this construction can only construct matrices of order 2^k for k integer. Whilst this is restrictive on the number of image pixels one can obtain, this is not of big concern to us since many images are of $2^k \times 2^k$ size anyways. For us, a bigger concern is that when using this construction technique, the masks appear to have different spatial frequencies as can been in supplementary figure 4. When undersampling, one has to choose which masks to omit and removing the low frequency masks can have detrimental effects on the image. Selecting only the low-frequency masks has similar effects to just downsampling[6].

Fortunately, other construction techniques do exist. Namely, the *Paley type I Hadamard construction technique*[7]. Let q be a prime power congruent to $3 (\mod 4)$. Then one constructs a vector, V, of length q in which the ith entry equals $\chi(i-1)$, where

$$\chi(x) = \begin{cases} 0 & \text{if } x = 0 \\ 1 & \text{if } x \text{ is a quadratic residue of } q \\ -1 & \text{if } x \text{ is a quadratic non-residue of } q. \end{cases} \tag{11}$$

Then one constructs a $q \times q$ matrix, Q, where the jth row is obtained by cyclically permuting V to the right $j-1$ times. Finally,

$$H_{q+1} = I + \begin{bmatrix} 0 & J^T \\ -J & Q \end{bmatrix}, \tag{12}$$

where J is a column vector of length q where every entry equals 1 and I is the identity matrix. This construction offers masks that are cyclical in nature, in other words mask $j+1$ is just mask j but shifted to the right as can be seen in supplementary figure 4. This means that these masks all have similar spatial frequencies and hence there is no need to pay attention to which masks one omits in the undersampling process. However, we need to mention that, for a 50% sampling ratios, selecting every odd mask number will give better results compared to selecting the first 50% of the masks. This is true for all sampling ratios.

Supplementary Note 7: Hyperspectral imaging

The culmination of the ideas in this work, namely the modulation geometry, software processing and undersampling, pave the way for a rapid THz spectroscopic imaging system without incurring great cost or complexity. We demonstrate this by taking a THz movie with 32×32 resolution and 10 frames-per-second, shown in supplementary video 1, where we use [1 0] Paley-Hadamard masks switched at a $4kHz$ rate with 40% sampling ratio. The imaged object consists of 4 materials: air, metal, $210\mu m$ and $420\mu m$ thick plastic, and the movie shows how the THz pulse arrives at different points in time depending on which material the THz has propagated through. Five frames taken at different points in time are shown in supplementary figure 5**a**, and it can be seen that the different materials have different temporal and transmission effects on the THz radiation. As is customary with THz-TDS systems, with a reference measurement and Fourier transformation one can calculate the transmission and phase delay through each material[2] and these are shown in figs. 5**b** and 5**c** respectively. As expected in fig. 5**b** it can be seen that metal and air regions have a transmittance of around 0 and 1, respectively, whereas the two plastic regions have similar values, around 0.7. Further, the temporal delays through each region are 0, 1.1 and $2.2ps$, for the air, $210\mu m$ and $420\mu m$-thick plastic, respectively, whereas the metallic region has inconsistent phase

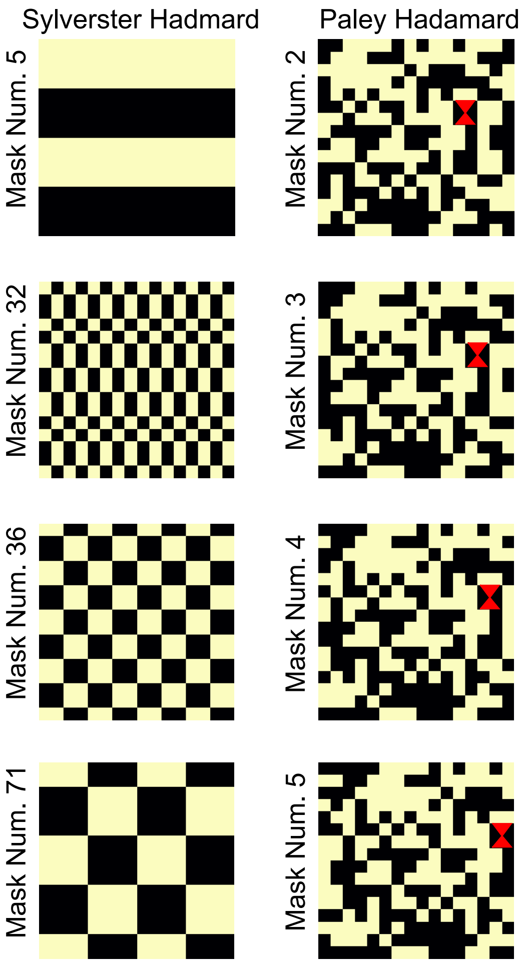

Supplementary Figure 4. Hadamard masks constructed by the Sylvester construction on the left, where we display mask numbers 5, 32, 36 and 71 in order to show the different 'spatial' frequencies of masks from this construction technique. On the right are four consecutive masks constructed via the Paley method, and the red triangles are only added as a visual guide to show how these masks are just horizontally shifted from one another.

values due to its low-transmission. This demonstrates that undersampling, when done correctly, reduces the measurement time without sacrificing image fidelity.

References

1. Hendry, E., Koeberg, M., Pijpers, J. & Bonn, M. Reduction of carrier mobility in semiconductors caused by charge-charge interactions. *Phys. Rev. B - Condens. Matter Mater. Phys.* **75**, 233202, DOI: 10.1103/PhysRevB.75.233202 (2007).

2. Ulbricht, R., Hendry, E., Shan, J., Heinz, T. F. & Bonn, M. Carrier dynamics in semiconductors studied with time-resolved terahertz spectroscopy. *Rev. Mod. Phys.* **83**, 543–586, DOI: 10.1103/RevModPhys.83.543 (2011).

3. Shrekenhamer, D., Watts, C. M. & Padilla, W. J. Terahertz single pixel imaging with an optically controlled dynamic spatial light modulator. *Opt. Express* **21**, 12507–12518, DOI: 10.1364/oe.21.012507 (2013).

4. Jeon, T.-I. & Grischkowsky, D. Nature of Conduction in Doped Silicon. *Phys. Rev. Lett.* **78**, 1106–1109, DOI: 10.1103/PhysRevLett.78.1106 (1997).

5. Sh. A. Furman & A. V. Tikhonravov. *Basics of optics of multilayer systems* (Frontière, Gif-sur-Yvette, 1992).

6. Sun, M. J., Meng, L. T., Edgar, M. P., Padgett, M. J. & Radwell, N. A Russian Dolls ordering of the Hadamard basis for compressive single-pixel imaging. *Sci. Reports* **7**, 3464, DOI: 10.1038/s41598-017-03725-6 (2017).

7. Paley, R. E. A. C. On orthogonal matrices. *J. Math. Phys.* **12**, 311–320, DOI: 10.1002/sapm1933121311 (1933).

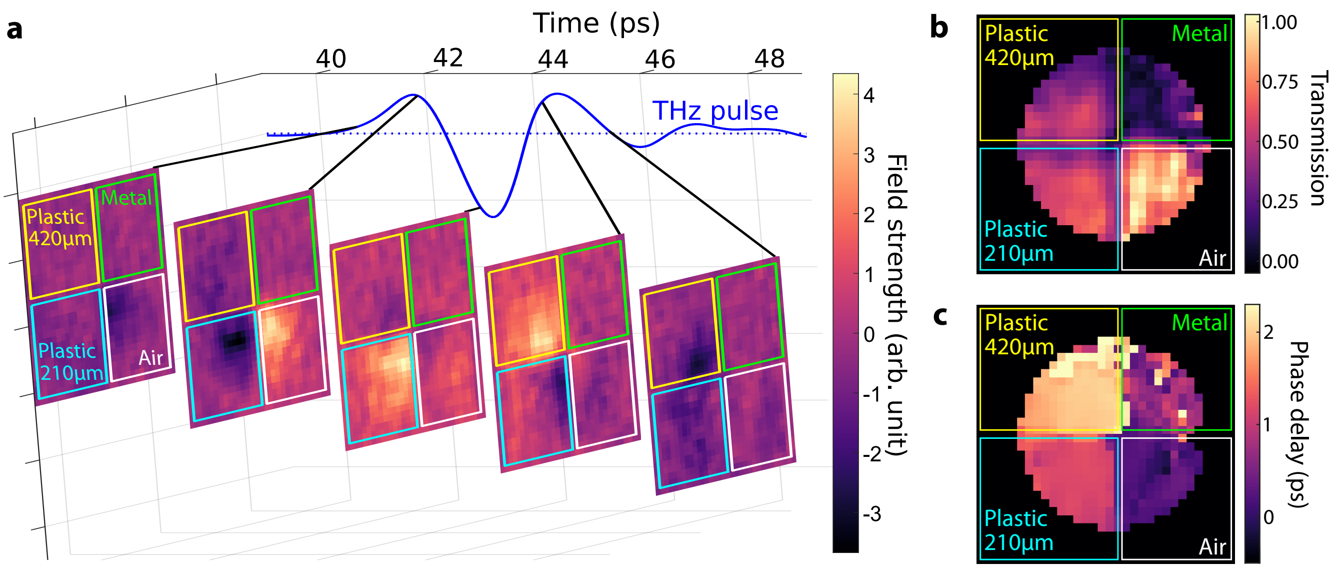

Supplementary Figure 5. **Terahertz movie frames. a:** Five frames of the Terahertz movie shown as supplementary video 1. The movie shows the real-time recording of a THz pulse as it propagates through four different materials: air, metal, 210μm and 420μm thick plastic. The displayed frames have been taken at different temporal points of the THz pulse, with solid black lines indicating the time. **b-c:** The transmission and phase delay through the four distinct regions as calculated from the THz movie.
