# Supplementary material for: Real-time terahertz imaging with a single-pixel detector
Source: Nat Commun. 2020 May 21;11:2535. doi: 10.1038/s41467-020-16370-x (PMC7242476; doi:10.1038/s41467-020-16370-x)
Supplement: Supplementary file 3 — Description of Additional Supplementary Files [file 41467_2020_16370_MOESM3_ESM.pdf]

Description of Additional Supplementary Files

File name: Supplementary Movie 1
Description: Real-time display of a THz image. A thin metal bar is placed in a THz beam with a Gaussian profile, and the resulting image is displayed on the monitor on the right. Red colour means strong field amplitude and blue means weak field amplitude. The THz video-stream is 32x32 resolution with about 6 frames-per-second, which is enough to see the metal bar move in real-time with the THz beam. We use [1 0] Paley-Hadamard masks switched at a 3.125kHz rate with 40% sampling ratio.

File name: Supplementary Movie 2
Description: A hyperspectral THz image. This is 32x32 THz hyperspectral image shown as a movie with 10 frames-per-second. We use [1 0] Paley-Hadamard masks switched at a 4kHz rate with 40% sampling ratio for image acquisition at each temporal point. The imaged object consists of 4 materials: air, metal, 210µm and 420µm thick plastic, and the movie shows how the THz pulse arrives at different points in time depending on which material the THz has propagated through.
